# Interpretation of individual differences in computational neuroscience using a latent input approach

**DOI:** 10.1016/j.dcn.2025.101512

**Published:** 2025-01-16

**Authors:** Jessica V. Schaaf, Steven Miletić, Anna C.K. van Duijvenvoorde, Hilde M. Huizenga

**Affiliations:** a Cognitive Neuroscience Department, Donders Institute for Brain, Cognition and Behaviour, Radboud University Medical Center, Nijmegen, the Netherlands; b Cognitive Psychology Unit, Institute of Psychology, Leiden University, the Netherlands; c Integrative Model-Based Cognitive Neuroscience Unit, Department of Psychology, University of Amsterdam, the Netherlands; d Developmental and Educational Psychology Unit, Institute of Psychology, Leiden University, the Netherlands; e Department of Developmental Psychology, University of Amsterdam, the Netherlands

**Keywords:** Computational neuroscience, Developmental neuroscience, Individual differences, Model selection

## Abstract

Computational neuroscience offers a valuable opportunity to understand the neural mechanisms underlying behavior. However, interpreting individual differences in these mechanisms, such as developmental differences, is less straightforward. We illustrate this challenge through studies that examine individual differences in reinforcement learning. In these studies, a computational model generates an individual-specific prediction error regressor to model activity in a brain region of interest. Individual differences in the resulting regression weight are typically interpreted as individual differences in neural coding. We first demonstrate that the *absence* of individual differences in neural coding is not problematic, as such differences are already captured in the individual specific regressor. We then review that the *presence* of individual differences is typically interpreted as individual differences in the use of brain resources. However, through simulations, we illustrate that these differences could also stem from other factors such as the standardization of the prediction error, individual differences in brain networks outside the region of interest, individual differences in the duration of the prediction error response, individual differences in outcome valuation, and in overlooked individual differences in computational model parameters or the type of computational model. To clarify these interpretations, we provide several recommendations. In this manner we aim to advance the understanding and interpretation of individual differences in computational neuroscience.

## Highlights

• Computational neuroscience elucidates neural underpinnings of behaviors.
• It is also used to study individual, for example developmental, neural differences.
• We demonstrate ambiguities in the interpretation of individual neural differences.
• We therefore provide several recommendations to disambiguate interpretations.

## 1 Introduction

Computational neuroscience offers valuable opportunities to understand the neural processes underlying a wide range of behaviors, including reinforcement learning, value-based decision-making, and delay discounting (Chase et al., 2015, Garrison et al., 2013, Humphreys et al., 2016, Lockwood and Klein-Flügge, 2021, Rangel et al., 2008, Trepel et al., 2005, Williams et al., 2021). These computational approaches provide unique advantages to understand the relation between cognitive theories, behavioral data and their neural mechanisms (e.g., Farrell and Lewandowsky, 2015).

In the last decade, the approach has also become prominent in the study of *individual differences* in neural processes (Patzelt et al., 2018). For example, the approach has been used to investigate developmental differences (reviews by Van den Bos et al., 2017; Van den Bos and Eppinger, 2016; DePasque and Galván, 2017; Hartley and Somerville, 2015; Hofmans and Van den Bos, 2022; Lourenco and Casey, 2013), learning differences (review by Van Duijvenvoorde et al., 2022), and differences related to mental illnesses (reviews by Hauser et al., 2019; Huys et al., 2016; Sonuga-Barke et al., 2016).

It has been argued that the study of individual differences by means of a computational neuroscience approach has two main advantages. First, the possibility to investigate individual differences in the neural coding of latent variables, that is underlying cognitive processes, instead of in the coding of manifest variables like stimuli or responses (Hartley and Somerville, 2015, Hauser et al., 2019, Huys et al., 2016, Patzelt et al., 2018, Van den Bos et al., 2017). Second, the formulation of computational models, which stimulates formal, instead of verbal, theories, makes the origins of individual differences explicit (Guest and Martin, 2021, Patzelt et al., 2018, Van den Bos et al., 2017).

There are multiple computational neuroscience approaches. A common approach is to fit a computational model to behavioral data, after which a latent variable derived from this model is entered as a predictor in the fMRI analysis. This so-called latent input approach (Turner et al., 2017) is the main topic of the current paper. Other approaches to computational neuroscience include fitting a computational model to the fMRI data directly (e.g., Ashby and Waldschmidt, 2008; Van Gerven, 2017) and fitting a computational model to behavioral and fMRI data simultaneously (e.g., Turner et al., 2013; Turner and Mcclure, 2015). A final computational neuroscience approach is to use machine learning, and not computational models, to predict individual differences from brain-related indices (e.g., Huys et al., 2016). We here focus on the first approach, and do not aim to generalize to the other three approaches.

Given the widespread use of the latent input approach, clear interpretation of individual differences in computational neuroscience studies is crucial. In this paper we illustrate that such interpretation is far from straightforward. We also provide recommendations to clarify interpretations, and if this is not feasible, to acknowledge the inherent ambiguity. In doing so, we summarize and extend previous methodological work on the interpretation of individual differences in neuroscience (Katahira and Toyama, 2021, Lebreton et al., 2019, Mumford et al., 2024, Poldrack, 2010). We aim to do so in a tutorial way, such that it is accessible for a wide audience interested in the study of individual differences by a computational neuroscience approach. We illustrate our line of reasoning by focusing on developmental differences in reinforcement learning, with a simple task and simple computational model. In the discussion, we argue that it is likely that scenarios and recommendations also apply to the study of other individual differences, for example related to mental health, other processes, like value-based decision making and intertemporal choice, and to more complex tasks and computational models.

## 2 Methods

We illustrate reasoning by focusing on computational neuroscience studies investigating individual, and particularly developmental, differences in reinforcement learning. To our knowledge, up to mid 2024, at least 12 developmental studies have used this approach (Boehme et al., 2017, Christakou et al., 2013, Cohen et al., 2010, Davidow et al., 2016, Hauser et al., 2015, Javadi et al., 2014, Jones et al., 2014, Rodriguez Buritica et al., 2024, Rosenblau et al., 2018, Van den Bos et al., 2012, Waltmann et al., 2023, Westhoff et al., 2021). These studies implemented a variety of reinforcement learning tasks and reinforcement learning models.

**Fig. 1:**
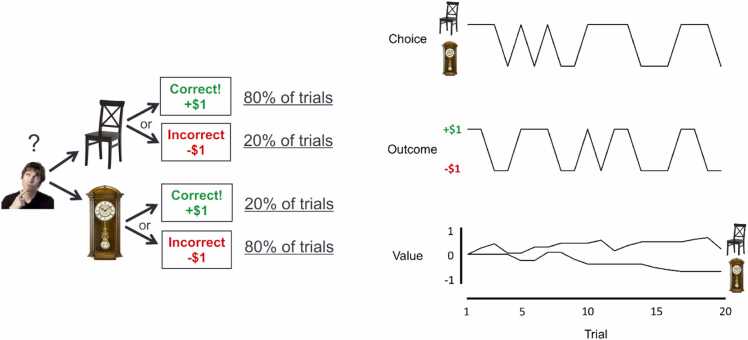
Example task with illustrative choice sequence. Left: Example trial in which a participant chooses between two stimuli that lead to probabilistic outcomes. Right: Example sequence of choices, experienced outcomes, and underlying value updating obtained from a reinforcement learning model.

For our purposes it suffices to describe a simple task (Fig. 1) in which participants repeatedly choose between two stimuli (e.g., a chair and a clock). After making a choice, the participant experiences an outcome (e.g., winning or losing one dollar) allowing them to gradually learn the value of each stimulus. This learned value then subsequently guides choice behavior in the next trial. Various computational models have been proposed to formalize the processes underlying these choices (for a review see Niv, 2009), one of which is inspired by the highly influential Rescorla-Wagner model (Rescorla and Wagner, 1972). We use this straightforward model to illustrate our line of reasoning, as it is sufficient for our current purposes.

A computational model can be fitted to the behavioral choice data of each individual *i* on trial *t*^2^. This model features a value (*V*) of the chosen option, which is defined by:

(1) $$V_{\mathrm{chair},i,t+1}=V_{\mathrm{chair},i,t}+\alpha_{i}\times {\mathrm{PE}}_{i,t}$$

where the prediction error (*PE*) is the difference between the observed outcome (*O*) and the value of the chosen option:

(2) $${\mathrm{PE}}_{i,t}=O_{i,t}-V_{\mathrm{chair},i,t}$$

The estimated probability of participant *i* choosing the chair over the clock on trial *t+1* is then given by the so-called softmax rule:

(3) $$\mathrm{Pr}\left(\mathrm{choic}e_{i,t}=\mathrm{chair}\right)=\frac{1}{1+e^{-\beta_{i}\left(V_{i,t,\mathrm{chair}}-V_{i,t,\mathrm{clock}}\right)}}$$

This model requires for each participant two parameters, the learning rate *α* and the inverse temperature *β.* If the learning rate is high, the outcome on a previous trial has a large effect on the estimated value. If the inverse temperature is high, choices are very much guided by differences in the values of the two stimuli.

The individual-specific prediction error variable can then be included as a first-level regressor into the functional Magnetic Resonance Imaging (fMRI) Generalized Linear Model (GLM) after convolution with a hemodynamic response function. That is, schematically and omitting convolution, the intercept and all other regressors:

(4) $$\mathrm{fMRI GLM}:\mathrm{neuralsigna}l_{i,t}=\phi_{i}\times {\mathrm{PE}}_{i,t}+\varepsilon_{i,t}$$

where the prediction error is defined as in Eq. 2, and ε refers to noise. The parameter $\phi_{i}$ then indicates for individual *i* the ‘neural coding’ of the prediction error in the brain. Specifically, it determines the scaling of the prediction errors to the neural signal.

This neural coding parameter $\phi_{i}$ is a common target for inferences on individual differences (e.g., Lebreton et al., 2019). For example, it is tested whether it relates to continuous individual difference characteristics, like age, or to nominal ones, like age group. The most common and most literal interpretation of individual differences in $\phi_{i}$ is that individuals differ in their neural encoding (Lebreton et al., 2019). If one participant has a higher neural coding parameter, this could indicate something ‘positive’ about this participant (e.g., the participant was more engaged in the task) or ‘negative’ (e.g., the participant required more neural resources and was therefore less efficient in carrying out the computations; Lebreton et al., 2019; Poldrack, 2010).

However, interpretation of the $\phi_{i}$ parameter, and individual differences in it, is more complex than it may initially seem. In the current paper, we focus on potential ambiguities in the interpretation of individual differences in computational neuroscience studies. While other, non-computational, factors may also affect the interpretation of these individual differences (Dubois and Adolphs, 2016, Poldrack et al., 2017), our focus here is specifically on computational factors.

We illustrate our reasoning through seven scenarios. In each scenario we provide recommendations on how to clarify interpretations, and when this is not feasible, a recommendation on how to report explicitly that interpretation remains ambiguous. In these scenarios we distinguish between three concepts: the prediction error obtained from modeling the behavioral choice data (a latent variable); the neural response observed in the brain (directly measured data); and the neural coding parameter $\phi_{i}$ estimated from the fMRI GLM, which describes the relation between the neural responses and the prediction errors for individual *i.* Our goal is merely to make researchers aware of interpretational challenges and to provide recommendations for addressing potential ambiguities. We therefore do not elaborate on potential interpretational problems in the studies mentioned above. In the discussion, however, we do use examples from these prior studies to illustrate the decisions researchers make relating to the seven scenarios.

## 3 Scenarios

The scenarios we present here are not intended to be exhaustive but they do represent common situations that may be encountered when interpreting individual differences in the neural coding parameter. We begin with five scenarios in which an adequate computational model was fitted to the behavioral choice data, and then proceed with two scenarios in which this was not the case.

### 3.1 Adequate computational model scenario 1: Lack of individual differences in neural coding

A perhaps intuitive interpretation suggest that if there are individual differences in the neural signal, these must be reflected in the $\phi_{i}\;$ parameter. However, individual differences can be *present* in the neural signal but *absent* in $\phi_{i}$. Referring to the fMRI GLM in Eq. 4, individual differences in the neural signal can arise if prediction errors differ between individuals, despite no differences in $\phi_{i}.\;$

Suppose one individual barely uses prediction errors to update estimated values, that is, uses a low learning rate of α = 0.05, whereas another does, that is, α = 0.5. Both choose the same option which has an estimated value of 0.5 and they get rewarded by 1 for choosing that option. Following Eq. 2, they both have a prediction error of 1 – 0.5 = 0.5. Yet, following Eq. 1, the updated value of the individual with the low learning rate becomes 0.5 + 0.05 * (1 – 0.5) = 0.525 and of the individual with the high learning rate 0.5 + 0.5 * (1 – 0.5) = 0.75. If they then again get rewarded by 1 for choosing that option, their prediction errors will differ because of different estimated values. Specifically, the first individual’s prediction error becomes 1 – 0.525 = 0.475, whereas those of the latter becomes 1 – 0.75 = 0.25. In the long run, prediction errors of the individual with the low learning rate will only deviate slightly (0.5 versus 0.475), resulting in small prediction error variance, whereas those of the individuals with the high learning rate (0.5 versus 0.25) will vary more.

**Fig. 2:**
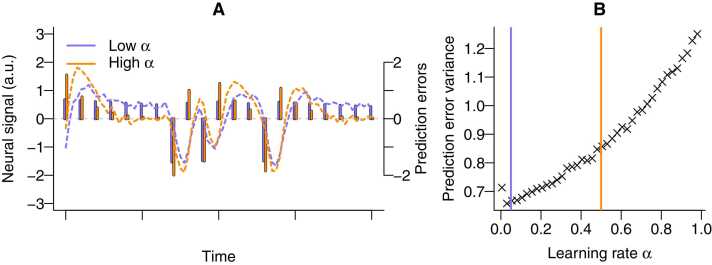
Lack of individual differences in neural coding. Differences in learning rates α (low = 0.05 versus high = 0.5) lead to differences in behavioral prediction errors and differences in neural signal, despite having the same neural coding parameter ϕ. Vertical sticks in panel A represent prediction errors for a simulated participant with a low and high learning rate, respectively. The dotted lines are simulated neural responses, obtained with Eq. 4 using a small amount of noise (SD = 0.1) for illustrative purposes. To ease visualization, we simulated a learning process without choice behavior (cf. classical conditioning), which ensures that both simulated participants received the same outcomes; as such, the differences in the prediction errors solely arise due to differences in learning rates in this example figure (see Fig. 7 A for a scenario with choice behavior). Differences in the neural signal arise due to differences in how quickly the size of the reward prediction error changes across trials, which can be quantified with their variance. Panel B shows the prediction error variance (mean across 50 simulated datasets) as a function of the learning rate.

Fig. 2 illustrates this point by showing how differences in learning rates lead to differences in behavioral prediction errors. As shown by the height of the vertical sticks, people with low learning rates (Fig. 2 A) adjust slower to the observed outcomes compared to people with higher learning rates (Fig. 2B), which leads to less variable behavioral prediction errors across the experiment. When we simulate the neural signal for both participants using Eq. 4 and use the same $\phi_{i}$ parameter for both participants, the resulting neural signal also differs between participants. Specifically, the more variable behavioral prediction errors for the participant with the high learning rate, translate into a more variable neural signal (Fig. 2 C). In other words, *because* the prediction errors are more variable, the neural signal is more variable as well – even when the $\phi_{i}$ parameter is the same for both participants.

As such, an absence of individual differences in $\phi_{i}\;$ cannot be interpreted as reflecting no individual differences in the neural signal. Rather, the individual differences in neural data are entirely captured by individual differences in the behavioral prediction error. Key point here is that the parameter $\phi_{i}$ does not reflect the size of a neural response. Rather, it reflects the size of the neural response *relative to* the size of the prediction errors.

In terms of development, this absence of age-related individual differences in neural coding $\phi_{i}$ does not necessarily imply an absence of age-related differences in neural responses. Instead, it indicates that the *scaling* of behavioral prediction errors to neural responses is consistent across participants.

This scenario illustrates that individual differences in behavioral prediction errors do not necessarily co-occur with such differences in the neural coding of these prediction errors. This may be perceived as counterintuitive as, in many cases of cognitive neuroscience, researchers expect individual differences in behavioral data to co-occur, or even result from, individual differences in neural data (e.g., Kanai and Rees, 2011; Marek et al., 2022). The same holds for some other computational neuroscience approaches like the two-stage neural approach, in which behavioral and fMRI model parameters are expected to covary (Forstmann et al., 2008). To explicate these expectations, our recommendation therefore is to explicitly state in a pre-registration whether, given the theoretical framework and specific research question, individual differences are expected in behavioral prediction errors, neural coding, or in both, and whether it is expected that behavioral and neural individual differences correlate.

### 3.2 Adequate computational model scenario 2: Spurious individual differences in neural coding due to standardization of prediction errors

Individual differences in neural coding may arise due to how behavioral prediction errors are included in the fMRI GLM. Specifically, standardization, that is, dividing prediction errors by their standard deviation before entering them as regressors may lead to individual differences in the $\phi_{i}$ parameter. That is, if we divide the prediction error by an individual-specific standard deviation $sd_{i}$, the $\phi_{i}$ parameter is necessarily multiplied by this individual-specific standard deviation (cf. Eq. 4), see also Fig. 2b in Lebreton et al. (2019):

(5) $$\mathrm{fMRI GLM}:\mathrm{neuralsigna}l_{i,t}=\left(\phi_{i}\times sd_{i}\right)\times \left({\mathrm{PE}}_{i,t}/sd_{i}\right)+\varepsilon_{i,t}$$

If there are no individual differences in $\phi_{i}$, individual differences in the neural coding parameter, now $\left(\phi_{i}\times sd_{i}\right)$, can be considered as spurious, as they are due to individual differences in the standard deviation of the behavioral prediction error (Lebreton et al., 2019). Statistically, standardization may be a useful tool, for example, to put all regressors on a similar scale. However, substantively it is implausible that the neural response reflects ‘standard deviation adjusted prediction errors’ in exactly the way they are modeled, for the simple reason that determining this standard deviation requires participants’ knowledge of all prediction errors, which can only be known after the experiment is complete.

This scenario is also pertinent for developmental studies, as learning rates may differ between younger and older age groups (see Nussenbaum and Hartley, 2019 for a review). As shown in scenario 1, this will lead to age-related differences in the variability, and thus standard deviation, of prediction errors (see Fig. 2). Therefore, standardizing behavioral prediction errors in the fMRI GLM is likely to result in spurious age-related differences in neural coding.

Therefore, we propose two recommendations to address scenario 2. First, to avoid these spurious individual differences, we recommend using unstandardized prediction errors in the fMRI GLM. Second, it is advised to report whether behavioral prediction errors were standardized or not, for example using the Cobidas checklist (https://osf.io/uvfr4). This allows readers to assess whether standardization could be a potential source of individual differences.

### 3.3 Adequate computational model scenario 3: Individual differences in neural coding originate in individual differences in the use of brain resources

In scenario 1, we showed that individual differences in neural responses may arise due to individual differences in behavioral prediction errors, even in the absence of individual differences in neural coding. In the current scenario 3, we build on this scenario by also including individual differences in the neural coding parameter $\phi_{i}$.

**Fig. 3:**
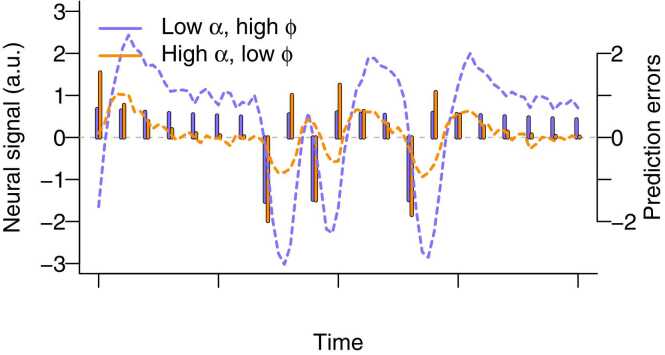
Presence of individual differences in neural coding. Differences in learning rates α (low = 0.05 versus high = 0.5) lead to differences in prediction errors and differences in neural signal, which can co-occur with differences in neural coding ϕ (low = 0.5 versus high = 2). Vertical sticks represent prediction errors for a simulated participant with a low learning rate and high neural coding in purple, and a high learning rate and low neural coding in orange. The dotted lines are simulated neural responses, obtained with Eq. 4 using a small amount of noise (SD = 0.1) for illustrative purposes. Both simulated participants received the same outcomes; as such, the differences in the prediction errors solely arise due to differences in learning rates. Contrary to Fig. 2, the neural responses of the participant with the low learning rate are stronger.

To illustrate this, we extend the scenario 1 example by including a high $\phi_{i}\;$ parameter for the participant with the lower learning rate, and vice versa, a low $\phi_{i}$ parameter for the participant with the high learning rate. Thus, participants differ in both learning rate and in neural coding. As illustrated in Fig. 3, the combination of these parameters results in a markedly different neural response from the response compared to that in Fig. 2, with now a *more* variable neural signal for the participant with the lower learning rate, driven by the larger $\phi_{i}$.

Individual differences in prediction errors may thus co-occur with individual differences in neural coding. This highlights the added value of fMRI: as it provides additional insights into effects not observable in behavior alone. Our first recommendation is again to explicate expectations regarding individual differences in behavioral prediction errors, neural coding, or both in a pre-registration. The second recommendation relates to the possibility that individual differences in a region of interest may also reflect individual differences in the brain regions where prediction errors are coded. That is, a diminished coding in a region of interest could simply indicate that the prediction error is coded in another brain region. We therefore recommend to also pre-register whether it is expected that individual differences in a region of interest, or in spatial organization, are expected. In the former, it suffices to test individual differences in the ROI. In the latter, it is recommended to pre-register how the individual differences in spatial organization will be tested, in which several approaches can be adopted. For instance, one may test multivariate whether there are individual differences in multiple ROIs (Fornari et al., 2023, Speer et al., 2023) or one may perform a whole-brain analysis to test individual differences in the entire brain.

### 3.4 Adequate computational model scenario 4: Spurious individual differences in neural coding due to neglected individual differences in the duration of neural responses

**Fig. 4:**
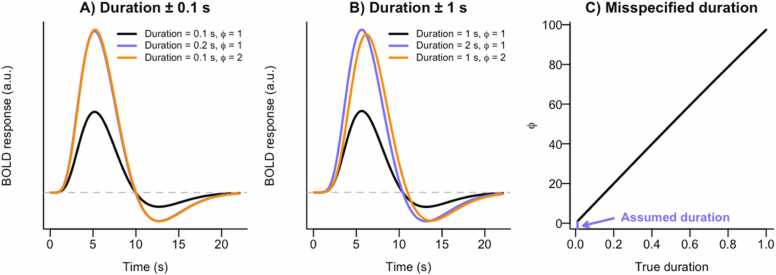
Duration of neural responses. When the duration of the neural response is relatively short (A: ± 0.1 second, B: ± 1 second), the effect of doubling the duration on the BOLD response is highly similar to the effect of doubling the neural coding parameter ϕ. This implies that any observed increase in ϕ could have been caused by an increase in duration. Panel C illustrates the near-linear relationship between ϕ and duration: when the duration is assumed to be 0.01 second (i.e., a stick function), but the true duration is larger (e.g., up to 1 second), ϕ is overestimated to an extent that is nearly linearly related to the size of misspecification of the duration.

Individual differences in neural coding may arise if individual differences in the duration of a response are overlooked. As illustrated in Fig. 4 (see also Fig. 1 in Mumford et al., 2023), a response lasting twice as long generates a neural signal nearly identical to that of a response twice as strong (Mumford et al., 2023).

The first recommendation for addressing this scenario is to account for the duration of the neural response in the neural model. This can be done by including events with individual-, or even trial-, specific durations instead of assuming a constant duration such as a stick function (Mumford et al., 2023). This approach is relatively straightforward when the duration of a neural signal can be observed, for example, via reaction times (Carp et al., 2012, Mumford et al., 2023). However, when this duration is not directly observable, for example, in case of a prediction error signal in the brain, it becomes more challenging. There are methods to estimate the duration of the hemodynamic response function (Lindquist et al., 2009, Lindquist and Wager, 2007), however they have only been applied to responses presumed to be longer in duration than prediction error signals.

We realize that accounting for the duration of the neural response is easier said than done. Therefore, the final recommendation for addressing interpretational ambiguities in this scenario is to acknowledge in the discussion of a paper that observed individual differences in $\phi_{i}$ may stem not only from individual differences in neural coding, but also from individual differences in the duration of the neural response.

There likely exist developmental differences in the duration of responses. For example, reaction times decrease from childhood to adulthood (Huizinga et al., 2006, Ratcliff et al., 2012). Such age-related decreases in the duration of responses may thus introduce spurious age-related decreases in the neural coding parameter $\phi_{i}$.

### 3.5 Adequate computational model scenario 5: No spurious individual differences in neural coding due to individual differences in inverse temperature

In Fig. 2 we showed that individual differences in the learning rate *α* lead to individual differences in the neural response, but not in the neural coding parameter *ϕ*. In this section and the next, we focus on the effect of the inverse temperature parameter *β*. Do individual differences in this parameter introduce individual differences in neural coding? The short answer is no, as explained further below.

**Fig. 5:**
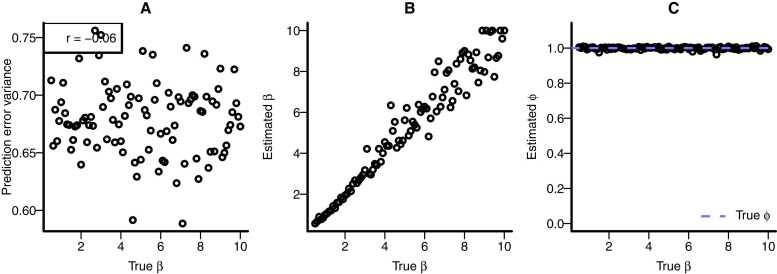
Inverse temperature. The effect of simulated inverse temperature settings on prediction error variance (A), the estimated inverse temperature β (B), and the estimated neural coding parameter ϕ (C). Each point represents a simulated dataset of 1000 trials, a simulated ”true” learning rate α = 0.1, and the simulated “true” inverse temperature β on the x-axis. Simulations assumed the choice paradigm depicted in Fig. 1, where outcomes were generated dependent on the choice. Neural data were simulated with a GLM with a neural coding parameter ϕ = 1. After simulating, a reinforcement learning model was fitted to the simulated behavioral data, and ϕ was estimated from the neural data using the fMRI GLM with the estimated prediction errors as predictor. It can be seen there is no systematic relationship between β and the prediction error variance (A), β can be accurately recovered from the behavioral data (B) but does not influence the estimated value of the neural coding parameter ϕ (C), which is also estimated accurately across a wide range of values of β.

The inverse temperature parameter *β* in Eq. 3 indicates how differences in values are weighted in the choice process. That is, if two participants both have a high value estimate for ‘chair’ and a low value estimate for ‘clock’, but differ in their inverse temperature, the participant with the higher inverse temperature is more likely to choose ‘chair’ than the other participant. The inverse temperature does not weigh prediction errors. It follows that when two participants have different inverse temperatures, no systematic differences in prediction errors are expected. Moreover, there is no reason to expect that different inverse temperatures will affect neural coding. A simulation (Fig. 5) confirms this: when simulating the simple RL paradigm depicted in Fig. 1 and (1), (2), (3), (4) (with 1000 trials) using a wide range of inverse temperatures, we observe no systematic relation between inverse temperature *β* and prediction error variance. While *β* can be accurately estimated from the behavioral data, it does not influence the estimated neural coding parameter *ϕ.*

In terms of development, this result has important implications. It has been shown that inverse temperature typically decreases with age (Nussenbaum and Hartley, 2019). The aforementioned result thus indicates that potential age-related differences in neural prediction error coding were not spuriously introduced by age-related differences in inverse temperature. Our general recommendation regarding this scenario thus is simple: it does not matter.

This result depends critically on the assumption that individual differences in the inverse temperature parameter solely reflect differences in choice behavior. However, individual differences in the inverse temperature can also reflect individual differences in outcome sensitivity, which has markedly different implications, as we detail in the next scenario.

### 3.6 Inadequate computational model scenario 6: Spurious individual differences in neural coding due to neglected individual differences in outcome sensitivity

We now turn to two scenarios in which the computational model is inadequately specified. In scenario 6, we address the case where individual differences in outcome sensitivity are not adequately modeled.

For example, one participant may be more sensitive to outcomes of $1 and -$1 than others (e.g., Fontanesi et al., 2019; Pedersen et al., 2017). A straightforward way to implement this outcome sensitivity in the computational model is to introduce a third individual-specific outcome sensitivity parameter, denoted as $\gamma_{i}\;$, to Eq. 2 (Ahn et al., 2008, Huys et al., 2013, Steingroever et al., 2014):

(6) $${\mathrm{PE}}_{i,t}=\gamma_{i}\times O_{i,t}-V_{\mathrm{chair},i,t}$$

**Fig. 6:**
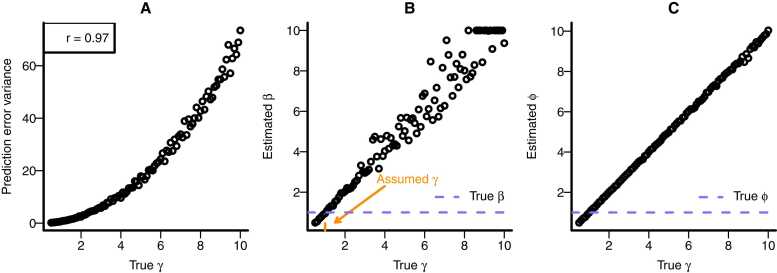
Outcome sensitivity. The effect of simulated outcome sensitivity on true prediction error variance (A); estimated inverse temperature β in case of a model misspecification (B), and the estimated neural coding parameter ϕ in case of a model misspecification (C). Each point represents a simulated dataset of 1000 trials, simulated with a “true” learning rate of α = 0.1, a “true” inverse temperature of β = 1, and a “true” outcome sensitivity γ depicted on the x-axis. Neural data were simulated with a GLM with a neural coding parameter ϕ = 1. After simulating, a reinforcement learning model was fitted to the simulated behavioral data. The model made the incorrect assumption that output sensitivity γ was 1 and estimated both the inverse temperature (B) and neural coding (C). The figures demonstrate that outcome sensitivity influences prediction error variance (A). Incorrectly assuming that individual differences in outcome sensitivity are absent introduces spurious individual differences in the estimated β parameter (B), and spurious individual differences in the neural coding parameter ϕ (C).

From Eq. 6, it is clear that the outcome sensitivity parameter γ influences prediction errors. Simulations (see Fig. 6 A) confirm that outcome sensitivity affects prediction error variance. As a result, if we fail to adequately model individual differences in outcome sensitivity, we will introduce spurious individual differences in the neural coding parameter $\phi_{i}$ (see Fig. 6 C).

A perhaps intuitive suggestion for handling scenario 6 is to incorporate individual differences in outcome sensitivity in the computational model. However, this is challenging, because individual differences in outcome sensitivity lead to exactly the same predictions about choice behavior as individual differences in inverse temperature (see also Katahira and Toyama, 2021). This was already foreshadowed in Fig. 6B: neglecting individual differences in outcome sensitivity introduces individual differences in the inverse temperature. This can also be shown formally, as we will do next.

With some algebra, it can be shown that the outcome sensitivity parameter γ is formally equivalent to the inverse temperature parameter *β*. That is, weighing all outcomes by a factor γ effectively leads to all *value estimates* being weighed by that factor γ(e.g., Van Maanen and Miletić, 2021). As a result, Eq. 3 can be rewritten as:

(7) $$\mathrm{Pr}\left(\mathrm{choic}e_{i,t}=\mathrm{chair}\right)=\frac{1}{1+e^{-\beta_{i}\left(\gamma_{i}\times \mathrm{valu}e_{i,t,\mathrm{chair}}-\gamma_{i}\times \mathrm{valu}e_{i,t,\mathrm{clock}}\right)}}=\;\frac{1}{1+e^{-\beta_{i}\times \gamma_{i}\left(\mathrm{valu}e_{i,t,\mathrm{chair}}-\mathrm{valu}e_{i,t,\mathrm{clock}}\right)}}$$

From the equation it can be derived that increases in γ have the same effect as decreases in *β*, and vice versa, when fitting the model to the same dataset. This may seem to contradict the simulation results in Fig. 6B which show a positive relationship between γ and *β.* Yet, this figure illustrates a different case, namely what happens to the estimated inverse temperature when assuming an outcome sensitivity of 1 that is truly different. In this case, any variability that results from true individual differences in outcome sensitivity loads onto the inverse temperature parameter.

This implies that individual differences in outcome sensitivity have *exactly* the same effect as individual differences in choice behavior.^3^ Therefore, comparing a model without and with individual differences in outcome sensitivity does not yield information to decide whether individual differences are caused by inverse temperature settings or by outcome sensitivity. That is, one does not know whether one is in scenario 5 or in scenario 6. This is unfortunate, as scenario 5 will *not* introduce spurious individual differences in neural coding, whereas scenario 6 does.

Therefore, we recommend addressing scenario 6 by assessing individual differences in outcome sensitivity through external means. For example, this can be done with an outcome sensitivity questionnaire (Carver and White, 1994, Torrubia et al., 2001) or by using a number line estimation task (Siegler and Opfer, 2003). In the computational model, objective outcomes can then be replaced with their subjective counterparts. Alternatively, it may be considered to also analyze response times (e.g., Miletić et al., 2020). For example, it could be hypothesized that people with a high outcome sensitivity make faster decisions to quickly earn outcomes, whereas people with a high tendency to exploit, and thus a larger inverse temperature, carefully deliberate choice options, which may result in slower decisions. Third, it has been suggested to replace monetary outcomes (cf. Fig. 1) with points, as it may be less likely that there are individual, for example developmental, differences in the valuation of points than in the valuation of money (Geier and Luna, 2012, Teslovich et al., 2014). However, to our knowledge no studies tested whether this is actually the case (see e.g., the review in Kray et al., 2018). Moreover, if money is replaced by points, care should be taken to not generalize to money-based paradigms, as generalization is something which should be tested and cannot be assumed (Dekay et al., 2022, Eckstein et al., 2022, Yarkoni, 2022). If it is not feasible to incorporate any of these recommendations, we advise to state explicitly in the discussion of a study that individual differences in neural coding may have been introduced by individual differences in outcome sensitivity.

### 3.7 Inadequate computational model scenario 7: spurious individual differences in neural coding due neglecting individual differences in the computational model

In scenario 6 we discussed the case where the computational model did not account for individual differences in its parameters, that is, in outcome sensitivity. We showed that these individual differences in computational model parameters could not be detected through model comparison and therefore could introduce spurious individual indifferences in neural coding. We now turn to other instances of inadequate modeling of individual differences in the computational model that may also introduce individual differences in neural coding. In some cases, such misspecification can be detected by computational model comparison. The role of model comparison in computational neuroscience studies has been investigated before (Katahira and Toyama, 2021, Wilson and Niv, 2015). Here, we add to this literature by showing its importance in the study of individual differences.

**Fig. 7:**
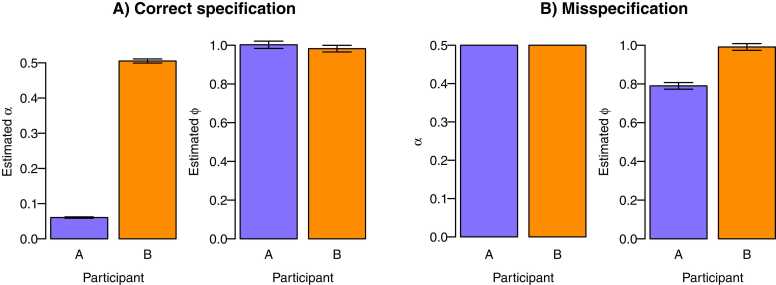
Individual differences in computational model parameters. When the fMRI GLM is fitted using the learning rates α that were estimated based on the behavioral data in scenario 1, one reaches the correct conclusion that ϕ was the same for both subjects (A). However, if the same learning rate α is incorrectly assumed for both individuals, one would conclude that individual differences exist in the neural coding parameter ϕ (B). fMRI simulations were done based on 300 trials, trial duration of 4 seconds, TR = 1, and a contrast-to-noise ratio of 0.1. A total of 400 datasets were simulated, and error bars indicate standard error across datasets. These small error bars indicate it is feasible to detect spurious individual differences in the neural coding parameter due to misspecification of learning rates.

As we already showed in scenario 1, individual differences in learning rates lead to differences in prediction error variance, which do not necessarily have to co-occur with individual differences in neural coding. We here illustrate that one may induce spurious individual differences in neural coding when one does not account for such differences in parameter estimates between individuals. First, consider a situation in which one individual has a low learning rate and the other a high learning rate (such as in scenario 1), but we assume the same high learning rate for both individuals. We compute the resulting prediction errors and use these as a predictor in the fMRI GLM. As illustrated in Fig. 7, we would incorrectly find individual differences in $\phi_{i}$ and thus conclude that participants differ in their neural coding.

Second, consider the more general situation in which individuals differ in the *type* of computational model that best describes their behavioral choice data. There is a wide variety of reinforcement learning models (e.g., Niv, 2009), and thus many potential sources of misspecifications. We illustrate one potential origin of misspecification here. Suppose some individuals are best described by the Rescorla-Wagner model in (1), (2), (3). This model features a static learning rate that does not change during the experiment. Other individuals, however, may be best described by a model in which the learning rate decreases during the experiment, because they become more confident and therefore learn less. The latter situation is for example modeled in a Pearce-Hall model (e.g., Roesch et al., 2012), a Bayesian learner model (e.g., Jepma et al., 2020), or with a decay function (e.g., Schaaf et al., 2022).

**Fig. 8:**
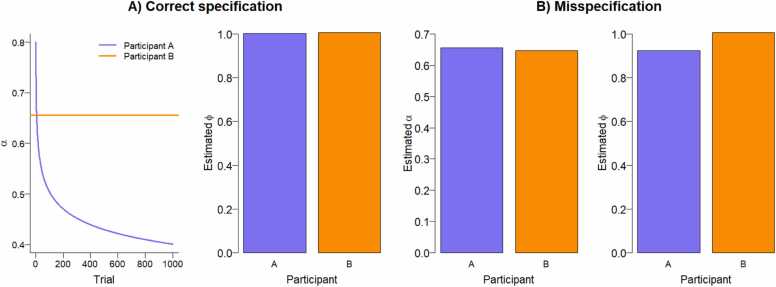
Individual differences in type of computational model. When the fMRI GLM is fitted using prediction errors from computational models that differ between participants, one reaches the correct conclusion that the neural coding ϕ was the same for both participants (A). However, if the same computational model was assumed for both participants, that is, if it was assumed that both participants used a static learning rate α, one would conclude that individual differences exist in the neural coding parameter ϕ (B).

What happens if data are generated with a dynamic, decaying learning rate but we incorrectly assume a static one following from the Rescorla-Wager model? As illustrated in Fig. 8B, the neural coding parameter *ϕ* will then be underestimated.^4^ This illustration thus shows that if one assumes there are no individual differences in the type of computational model, one may incorrectly conclude there are individual differences in neural coding.

Individual differences in the *type* of computational model, also known as strategy, are likely to be present. For example, developmental strategy differences have been observed in reinforcement learning (Crawley et al., 2020, Cutler et al., 2022, Jepma et al., 2020, Palminteri et al., 2016), reasoning (Jansen and Van der Maas, 2001, Lee and Sarnecka, 2011) and decision making (e.g. Huizenga et al., 2007; Huizenga et al., 2023; Jansen et al., 2012; Lang and Betsch, 2018; Mata et al., 2011).

This scenario of individual differences in the type of computational model can be addressed by model comparison. We must note, however, that adequate model comparison is by no means straightforward (Palminteri et al., 2017, Van den Bos et al., 2017). First, a large set of potential models should be compared because model comparison only tells you which model *in your model set* fits the data best, not whether you found the true underlying model. For instance, in a different context a staggering total of 70 models were compared (Valton et al., 2024). Second, it is crucial to determine whether the task and design allow to delineate these models (Huizenga et al., 2012, Van den Bos et al., 2017, Wilson and Collins, 2019). This can be done for example by means of simulation (Palminteri et al., 2017, Van den Bos et al., 2017, Wilson and Collins, 2019). Note the conclusion of this analysis may be that the task, the design, or both should be adapted to be able to differentiate between models, see also (Wilson and Collins, 2019).

We recommend two ways to address model comparison, both firmly grounded in the work of Lee (Lee, 2011, Lee and Newell, 2011, Lee and Webb, 2005; see also Daw, 2011). First, for each participant, a variety of models can be compared using a Bayesian approach (for a developmental application see Steingroever et al., 2019). The parameters of the best-fitting model are then used to compute the prediction errors which are subsequently fed into the fMRI GLM. A potential drawback of this approach is that computational model parameters are estimated at the individual level, which may introduce instability of the estimates (Nilsson et al., 2011). This potential drawback can be addressed by using a second approach: a hierarchical Bayesian model-based mixture analysis (for developmental applications see e.g., Bartlema et al., 2014; Huizenga et al., 2023; Zadelaar et al., 2021).^5^ In this approach, each participant is assigned to their best-fitting strategy, resulting in strategy groups. The computational model parameters in each strategy group follow a hierarchical structure, for example, all learning rates in the Rescorla-Wagner strategy group are normally distributed with a to-be-estimated mean and standard deviation. This method thus does not require estimation of model parameters for each individual separately, but only at the strategy group level, increasing parameter stability (Nilsson et al., 2011).

Adoption of such model comparison approaches offers a solution to reduce spurious individual differences in neural coding. For related approaches to studying individual differences in strategy use, we refer to Van Duijvenvoorde et al. (2016), Gluth et al. (2014), Park et al. (2011), Peters et al. (2014), Venkatraman et al. (2009), and Zadelaar et al. (2019), see also Hayden and Niv (2021).

## 4 Discussion

A computational neuroscience approach to studying the origins of individual differences has gained increased popularity. For example, it has been applied to examine individual differences related to development, learning, and mental illnesses. However, potential challenges in the interpretation of individual differences from computational neuroscience studies have received less attention. To address this gap, we presented seven scenarios, all related to reinforcement learning, and provided recommendations on how to interpret individual differences in each scenario. Below we summarize scenarios and recommendations for future studies using a computational neuroscience approach to study developmental differences in reinforcement learning. We also indicate how these recommendations generalize to computational neuroscience studies of individual differences in other decision-making tasks. We would like to stress that multiple scenarios may occur simultaneously, and it is typically unclear which combination applies. Therefore, it may be helpful to implement all of the recommendations provided below.

First, we demonstrated that individual differences may emerge in behavioral prediction errors or in their neural coding, and that it is not necessary that these differences co-occur. This may sound counterintuitive as researchers may expect such co-occurrence or even that behavioral differences are *caused* by neural ones (e.g., Kanai and Rees, 2011; Marek et al., 2022). This intuition is also reflected in the developmental computational neuroscience literature pertaining to reinforcement learning with many explicitly mentioning expectations on co-occurrence. For instance, hypothesizing “that developmental differences in striatal sensitivity to rewards might contribute to the observed developmental differences in adaptive behavior” (Van den Bos et al., 2012) or “that inter-individual differences in learning behavior […] are associated with differences in PE-related activation” (Boehme et al., 2017). Our recommendation is to explicitly state in a pre-registration which behavioral and neural individual differences are expected, to also pre-register whether it is hypothesized these are correlated, and to report on all results pertaining to these expectations.

Second, we demonstrated that spurious individual differences in neural coding can be introduced by standardizing prediction errors in the fMRI analysis. Therefore, we recommend against standardizing. When reading the developmental computational literature, we realized it is uncommon (with some exceptions, e.g., Cohen et al., 2010; Rodriguez Buritica et al., 2024) to report whether standardization was used or not. It is therefore difficult to determine whether standardization causes interpretational problems in the existing literature. This brings us to an additional recommendation to explicitly report whether or not standardization was used.

Third, we showed that individual differences in neural coding are often attributed to differences in the extent to which brain resources are used. In the developmental computational literature these differences are typically investigated in ROIs and interpreted in terms of coding intensity (Boehme et al., 2017, Christakou et al., 2013, Cohen et al., 2010, Davidow et al., 2016, Hauser et al., 2015, Jones et al., 2014, Rodriguez Buritica et al., 2024, Rosenblau et al., 2018, Waltmann et al., 2023, Westhoff et al., 2021), not efficiency. These ROI analyses suffice for testing differences to be interpreted as coding intensity or efficiency, whereas multivariate analyses are required to test for individual differences in spatial organization. Our recommendation therefore is to pre-register whether univariate or multivariate individual differences are expected, and to report all results pertaining to these expectations.

Fourth, we demonstrated that neglected individual differences in the duration of neural responses will introduce spurious individual differences in neural coding. Although the discussion on the confounding duration effect in fMRI is not new (e.g., Grinband et al., 2008), we share the goal of Mumford et al. (2023) to revive the discussion around interpretational challenges that arise from this confound. We believe it common practice to fix the duration of the neural response to arbitrarily short (Grinband et al., 2008). This is also reflected in the reviewed developmental computational neuroscience studies in which the duration was fixed to one second (Javadi et al., 2014) or to infinitely small or a stick function (Cohen et al., 2010, Rodriguez Buritica et al., 2024, Van den Bos et al., 2012, Westhoff et al., 2021). Ideally, we recommend including the duration of the neural response in the neural model. Alternatively, if this is not feasible, which often will be the case, we recommend to state explicitly that individual differences in neural coding may have been introduced by unaccounted individual differences in response duration.

Fifth, we showed that individual differences in inverse temperature do not introduce spurious differences in neural coding. However, in a subsequent, sixth, scenario, we showed that computational model comparison cannot disentangle individual differences in inverse temperature and those in outcome valuation, where the latter *does* introduce spurious individual differences in neural coding. In the reviewed literature, many different types of outcomes are used, including money (Boehme et al., 2017, Waltmann et al., 2023), points (Davidow et al., 2016, Rodriguez Buritica et al., 2024, Van den Bos et al., 2012), social feedback (Jones et al., 2014), and product ratings (Rosenblau et al., 2018). Interestingly, Davidow et al. (2016) reported that they used the word ‘correct’ or ‘incorrect’ as outcomes, without monetary incentives, “to avoid confounds related to the motivational significance of monetary reward across age groups.” We are not aware of any literature studying age-related differences in the valuation of each of the aforementioned outcome types (but see a very recent unpublished study by Veselic et al. (2024), indicating age-related differences in valuation). A potential solution may be to include an outcome valuation parameter instead of an inverse temperature parameter in the behavioral computational model, as has been done in the developmental reinforcement learning literature (Boehme et al., 2017, Waltmann et al., 2023).^6^ However, as outlined in scenario 6, prediction errors will differ between an analysis using an outcome valuation parameter and one using an inverse temperature parameter, which in turn affects the neural coding. We showed that it is impossible to determine which of the two computational models–using inverse temperature or outcome valuation–captures the true underlying mechanism. This leads to our recommendation to explore by alternative means whether individual differences in valuation exist. If this proves to be infeasible, we recommended explicitly stating that neural coding differences may have arisen from unaccounted individual differences in valuation.

Finally, we demonstrated that neglecting individual differences in computational model parameters, or in the type of computational models may introduce spurious differences in neural coding. Our final recommendation, therefore, was to select individual-specific models with individual-specific, or at least age-group specific parameters. In our reading of the literature, we conclude many studies already implement this recommendation. Interestingly, one study (Hauser et al., 2015) did not include individual- or age-group specific parameters “because we were not interested to model any behavioral differences into fMRI regression analysis and in order to obtain canonical and stable parameter estimates”. Apart from individual-specific models and parameters, we wish to emphasize that it is also important to consider different models and to carefully select the considered model set (Palminteri et al., 2017, Van den Bos et al., 2017). Therefore, we also recommend to test through simulations or in pilot data whether the task and models allow one to delineate different behaviors and therefore different explanations (Huizenga et al., 2012, Van den Bos et al., 2017, Wilson and Collins, 2019).

A potential limitation of the current study is that we illustrated our reasoning with a simple task and a simple reinforcement learning model. It may be argued that the scenarios do not apply to other types of processes, tasks, or computational models. However, the scenarios we describe are not specific to a reinforcement learning context, nor are many of them tied to the specific task or model we used. In scenarios 1 and 3, we showed that individual differences in behavioral measures may or may not co-occur with such differences in neural coding. This is because the neural coding parameter captures the scaling of parametric modulators to the neural signal, a general characteristic of the latent input approach. Moreover, standardization of parametric modulators, as discussed in scenario 2, affects interpretation of the neural coding parameter regardless of the process of interest. The same applies to the impact of ignoring individual differences in the duration of neural responses (scenario 4). These points are thus specific to the latent input approach and remain relevant regardless of the process, task, and computational model under consideration. In addition, our finding that the inverse temperature parameter does not affect the interpretation of the neural coding parameter likely generalizes to all choice paradigms that are modeled with softmax. This includes, for instance, intertemporal choice tasks (where individuals choose between sooner-smaller, later-larger rewards) and risky decision tasks (where individuals choose between safe options with a high probability of low reward and risky options with a lower probability of higher reward). This finding is particularly relevant for developmental studies, where age-related differences in inverse temperature parameters are one of the most robust findings (Nussenbaum and Hartley, 2019, Topel et al., 2023). Finally, although we illustrated our points regarding inadequate model fitting (scenarios 6 and 7) using parameters from reinforcement learning models (i.e., outcome sensitivity and learning rate), parameters from other types of computational models that affect the variance of parametric modulators will likely induce spurious individual differences in neural coding if left unaddressed. It may also be argued that the scenarios do not apply to other types of individual differences, for example, related to mental health conditions (Hauser et al., 2019, Huys et al., 2016, Sonuga-Barke et al., 2016) or socio-economic status (Crone et al., 2024, Rakesh and Whittle, 2021). However, we do not see any reasons why the current scenarios would not generalize to these types of individual differences. Yet, we do recommend adapting the openly accessible code (available at https://osf.io/7shmn/) to check scenarios for other types of processes, tasks, and models.

Relatedly, we here focused on the most common methodology which assumes a single prediction error signal and corresponding neural coding per participant. A less-frequently used approach uses subtraction logic to decompose prediction errors into multiple components, such as a ‘baseline’ prediction error (in the absence of a manipulation) and an additive effect of a manipulation on the prediction error (see e.g., Eldar and Niv, 2015; Wittmann et al., 2008). In these approaches, both individual differences in the neural coding parameter of the baseline signal and of the additive signal could be of interest. While we expect scenarios presented in the current paper to be relevant for both types of neural coding parameters, future studies could adapt the methods presented here to rigorously investigate the implications of the seven scenarios on such approaches.

## 5 Conclusion

In conclusion, with this paper, we aim to provide computational neuroscientists with recommendations for uncovering the origins of individual differences, as interpreting these differences is often more complex than it may initially appear.

## Data statement

This ms only contains simulated data

## Funding

JVS was supported by a Hypatia fellowship from the Radboud UMC.

## CRediT authorship contribution statement

**Hilde Maria Huizenga:** Writing – review & editing, Writing – original draft, Supervision, Methodology, Conceptualization. **Anna CK van Duijvenvoorde:** Writing – review & editing. **Steven Miletić:** Writing – review & editing, Writing – original draft, Visualization, Software, Methodology, Formal analysis, Conceptualization. **Jessica Vera Schaaf:** Writing – review & editing, Writing – original draft, Visualization, Software, Methodology, Formal analysis, Conceptualization.

## Declaration of Competing Interest

The authors declare that they have no known competing financial interests or personal relationships that could have appeared to influence the work reported in this paper.

## Data Availability

No data was used for the research described in the article.

## Footnotes

2 Later, we discuss scenarios in which models are not fitted to each individual separately.

3 Note that in this scenario, gains and losses are multiplied by a common outcome sensitivity factor γ. However, if there is a differential sensitivity to gains and losses, for example, due to loss aversion, this loss aversion parameter can be estimated in addition to the inverse temperature parameter (Huizenga et al., 2023). Model selection may then indicate that a model with a loss aversion parameter better describes the data than a model without.

4 The extent to which φ is underestimated is related to the extent of misspecification. If learning rates only decrease slightly across the experiment, the estimated behavioral prediction errors from a dynamic learning rate model would be similar to those obtained with a static learning rate. In such a case, the effect of misspecification on the neural coding φ would be minimal.

5 Note the approach can also be used to assess strategy changes within each participant (Schaaf, Jepma, Visser, and Huizenga, 2019).

6 Actually, both studies included two outcome sensitivity parameters; one for positive and one for negative outcomes, but fixed learning rates over positive and negative prediction errors, whereas Boehme also allowed learning rates to differ.

## References

[bib1] Ahn W.Y., Busemeyer J.R., Wagenmakers E.J., Stout J.C. (2008). Comparison of decision learning models using the generalization criterion method. Cogn. Sci..

[bib2] Ashby F.G., Waldschmidt J.G. (2008). Fitting computational models to fMRI data. Behav. Res. Methods.

[bib3] Bartlema A., Lee M.D., Wetzels R., Vanpaemel W. (2014). A Bayesian hierarchical mixture approach to individual differences: case studies in selective attention and representation in category learning. J. Math. Psychol..

[bib4] Boehme R., Lorenz R.C., Gleich T., Romund L., Pelz P., Golde S., Beck A. (2017). Reversal learning strategy in adolescence is associated with prefrontal cortex activation. Eur. J. Neurosci..

[bib5] Carp J., Fitzgerald K.D., Taylor S.F., Weissman D.H. (2012). Removing the effect of response time on brain activity reveals developmental differences in conflict processing in the posterior medial prefrontal cortex. NeuroImage.

[bib6] Carver C.S., White T.L. (1994). Behavioral inhibition, behavioral activation, and affective responses to impending reward and punishment: the BIS/BAS scales. J. Personal. Soc. Psychol..

[bib7] Chase H.W., Kumar P., Eickhoff S.B., Dombrovski A.Y. (2015). Reinforcement learning models and their neural correlates: an activation likelihood estimation meta-analysis. Cogn., Affect. Behav. Neurosci..

[bib8] Christakou A., Gershman S., Niv Y., Simmons A., Brammer M., Rubia K. (2013). Neural and psychological maturation of decision-making in adolescence and young adulthood. J. Cogn. Neurosci..

[bib9] Cohen J.R., Asarnow R.F., Sabb F.W., Bilder R.M., Bookheimer S.Y., Knowlton B.J., Poldrack R.A. (2010). A unique adolescent response to reward prediction errors. Nat. Neurosci..

[bib10] Crawley D., Zhang L., Jones E.J.H., Ahmad J., Oakley B., Cáceres A.S.J., Loth E. (2020). Modeling flexible behavior in childhood to adulthood shows age-dependent learning mechanisms and less optimal learning in autism in each age group. PLOS Biol..

[bib11] Crone E.A., Bol T., Braams B.R., de Rooij M., Franke B., Franken I., Veenstra R. (2024). Growing Up Together in Society (GUTS): a team science effort to predict societal trajectories in adolescence and young adulthood. Dev. Cogn. Neurosci..

[bib12] Cutler J., Apps M.A.J., Lockwood P.L. (2022). Encyclopedia of the Human Brain.

[bib13] Davidow J.Y., Foerde K., Galván A., Shohamy D. (2016). An upside to reward sensitivity: the hippocampus supports enhanced reinforcement learning in adolescence. Neuron.

[bib14] Daw N.D. (2011). Trial-by-trial data analysis using computational models. Decis. Mak., Affect, Learn.: Atten. Perform. XXIII.

[bib15] Dekay M.L., Rubinchik N., Li Z., Boeck P.De (2022). Accelerating psychological science with metastudies: a demonstration using the risky-choice framing effect. Perspect. Psychol. Sci..

[bib16] DePasque S., Galván A. (2017). Frontostriatal development and probabilistic reinforcement learning during adolescence. Neurobiol. Learn. Mem..

[bib17] Dubois J., Adolphs R. (2016). Building a science of individual differences from fMRI. Trends Cogn. Sci..

[bib18] Eckstein M.K., Master S.L., Xia L., Dahl R.E., Wilbrecht L., Collins A.G. (2022). The interpretation of computational model parameters depends on the context. eLife.

[bib19] Eldar E., Niv Y. (2015). Interaction between emotional state and learning underlies mood instability. Nat. Commun..

[bib20] Farrell S., Lewandowsky S. (2015). An introduction to cognitive modeling. Introd. Model-Based Cogn. Neurosci..

[bib21] Fontanesi L., Gluth S., Spektor M.S., Rieskamp J. (2019). A reinforcement learning diffusion decision model for value-based decisions. Psychon. Bull. Rev..

[bib22] Fornari L., Ioumpa K., Nostro A.D., Evans N.J., De Angelis L., Speer S.P.H., Gazzola V. (2023). Neuro-computational mechanisms and individual biases in action-outcome learning under moral conflict. Nat. Commun..

[bib23] Forstmann B.U., Dutilh G., Brown S., Neumann J., Von Cramon D.Y., Ridderinkhof K.R., Wagenmakers E.J. (2008). Striatum and pre-SMA facilitate decision-making under time pressure. PNAS.

[bib24] Garrison J., Erdeniz B., Done J. (2013). Neuroscience and Biobehavioral Reviews.

[bib25] Geier C.F., Luna B. (2012). Developmental effects of incentives on response inhibition. Child Dev..

[bib26] Van den Bos W., Bruckner R., Nassar M.R., Mata R., Eppinger B. (2017). Computational neuroscience across the lifespan: promises and pitfalls. Dev. Cogn. Neurosci..

[bib27] Van den Bos W., Cohen M.X., Kahnt T., Crone E. a (2012). Striatum-medial prefrontal cortex connectivity predicts developmental changes in reinforcement learning. Cereb. Cortex.

[bib28] Van den Bos W., Eppinger B. (2016). Developing developmental cognitive neuroscience: from agenda setting to hypothesis testing. Dev. Cogn. Neurosci..

[bib29] Van Duijvenvoorde A.C.K., Figner B., Weeda W.D., Van der Molen M.W., Jansen B.R.J., Huizenga H.M. (2016). Neural mechanisms underlying compensatory and noncompensatory strategies in risky choice. *Journal of Cogntive*. J. Cognt. Neurosci..

[bib30] Van Duijvenvoorde A.C.K., Whitmore L.B., Westhoff B., Mills K.L. (2022). A methodological perspective on learning in the developing brain. Npj Sci. Learn..

[bib31] Gluth S., Rieskamp J., Büchel C. (2014). Neural evidence for adaptive strategy selection in value-based decision-making. Cereb. Cortex.

[bib32] Grinband J., Wager T.D., Lindquist M., Ferrera V.P., Hirsch J. (2008). Detection of time-varying signals in event-related fMRI designs. NeuroImage.

[bib33] Guest O., Martin A.E. (2021). How computational modeling can force theory building in psychological science. Perspect. Psychol. Sci..

[bib34] Hartley C.A., Somerville L.H. (2015). The neuroscience of adolescent decision-making. Curr. Opin. Behav. Sci..

[bib35] Hauser T.U., Iannaccone R., Walitza S., Brandeis D., Brem S. (2015). Cognitive flexibility in adolescence: Neural and behavioral mechanisms of reward prediction error processing in adaptive decision making during development. NeuroImage.

[bib36] Hauser T.U., Will G.J., Dubois M., Dolan R.J. (2019). Annual research review: developmental computational psychiatry. J. Child Psychol. Psychiatry Allied Discip..

[bib37] Hayden B.Y., Niv Y. (2021). The case against economic values in the orbitofrontal cortex (or anywhere else in the brain). Behav. Neurosci..

[bib38] Hofmans L., Van den Bos W. (2022). Social learning across adolescence: a Bayesian neurocognitive perspective. Dev. Cogn. Neurosci..

[bib39] Huizenga H.M., Crone E.A., Jansen B.R.J. (2007). Decision-making in healthy children, adolescents and adults explained by the use of increasingly complex proportional reasoning rules. Dev. Sci..

[bib40] Huizenga H.M., Wetzels R., van Ravenzwaaij D., Wagenmakers E.-J. (2012). Four empirical tests of unconscious thought theory. Organ. Behav. Hum. Decis. Process..

[bib41] Huizenga H.M., Zadelaar J.N., Jansen B.R.J., Olthof M.C., Steingroever H., Dekkers L.M.S., Agelink van Rentergem J.A. (2023). Formal models of differential framing effects in decision making under risk. Decision.

[bib42] Huizinga M., Dolan C.V., Van der Molen M.W. (2006). Age-related change in executive function: developmental trends and a latent variable analysis. Neuropsychologia.

[bib43] Humphreys K.L., Telzer E.H., Flannery J., Goff B., Gabard-Durnam L., Gee D.G., Lee S.S. (2016). Risky decision making from childhood through adulthood: contributions of learning and sensitivity to negative feedback. Emotion.

[bib44] Huys Q.J.M., Maia T.V., Frank M.J. (2016). Computational psychiatry as a bridge from neuroscience to clinical applications. Nat. Neurosci..

[bib45] Huys Q.J.M., Pizzagalli D.A., Bogdan R., Dayan P. (2013). Mapping anhedonia onto reinforcement learning: a behavioural meta-analysis. Biol. Mood Anxiety Disord..

[bib46] Jansen B.R.J., Van der Maas H.L.J. (2001). Evidence for the phase transition from rule I to rule II on the balance scale task. Dev. Rev..

[bib47] Jansen B.R.J., Van Duijvenvoorde A.C.K., Huizenga H.M. (2012). Development of decision making: sequential versus integrative rules. J. Exp. Child Psychol..

[bib48] Javadi A.H., Schmidt D.H.K., Smolka M.N. (2014). Adolescents adapt more slowly than adults to varying reward contingencies. J. Cogn. Neurosci..

[bib49] Jepma M., Schaaf J.V., Visser I., Huizenga H.M. (2020). Uncertainty-driven regulation of learning and exploration in adolescents: a computational account. PLOS Comput. Biol..

[bib50] Jones R.M., Somerville L.H., Li J., Ruberry E.J., Powers A., Mehta N., Casey B.J. (2014). Adolescent-specific patterns of behavior and neural activity during social reinforcement learning. Cogn., Affect. Behav. Neurosci..

[bib51] Kanai R., Rees G. (2011). The structural basis of inter-individual differences in human behaviour and cognition. Nat. Rev. Neurosci..

[bib52] Katahira K., Toyama A. (2021). Revisiting the importance of model fitting for model-based fMRI: it does matter in computational psychiatry. PLOS Comput. Biol..

[bib53] Kray J., Schmitt H., Lorenz C., Ferdinand N.K. (2018). The influence of different kinds of incentives on decision-making and cognitive control in adolescent development: a review of behavioral and neuroscientific studies. Front. Psychol..

[bib54] Lang A., Betsch T. (2018). Children’s neglect of probabilities in decision making with and without feedback. Front. Psychol..

[bib55] Lebreton M., Bavard S., Daunizeau J., Palminteri S. (2019). Assessing inter-individual differences with task-related functional neuroimaging. Nat. Hum. Behav..

[bib56] Lee M.D. (2011). How cognitive modeling can benefit from hierarchical Bayesian models. J. Math. Psychol..

[bib57] Lee M.D., Newell B.R. (2011). Using hierarchical Bayesian methods to examine the tools of decision-making. Judgm. Decis. Mak..

[bib58] Lee M.D., Sarnecka B.W. (2011). Number-knower levels in young children: insights from Bayesian modeling. Cognition.

[bib59] Lee M.D., Webb M.R. (2005). Modeling individual differences in cognition. Psychon. Bull. Rev..

[bib60] Lindquist M.A., Meng Loh J., Atlas L.Y., Wager T.D. (2009). Modeling the hemodynamic response function in fMRI: efficiency, bias and mis-modeling. NeuroImage.

[bib61] Lindquist M.A., Wager T.D. (2007). Validity and power in hemodynamic response modeling: a comparison study and a new approach. Hum. Brain Mapp..

[bib62] Lockwood P.L., Klein-Flügge M.C. (2021). Computational modelling of social cognition and behaviour - a reinforcement learning primer. Soc. Cogn. Affect. Neurosci..

[bib63] Lourenco F., Casey B.J. (2013). Neuroscience and Biobehavioral Reviews.

[bib64] Marek S., Tervo-Clemmens B., Calabro F.J., Montez D.F., Kay B.P., Hatoum A.S., Dosenbach N.U.F. (2022). Reproducible brain-wide association studies require thousands of individuals. Nature.

[bib65] Mata R., von Helversen B., Rieskamp J. (2011). When easy comes hard: the development of adaptive strategy selection. Child Dev..

[bib66] Miletić S., Boag R.J., Forstmann B.U. (2020). Mutual benefits: combining reinforcement learning with sequential sampling models. Neuropsychologia.

[bib67] Mumford J.A., Bissett P.G., Jones H.M., Shim S., Rios J.A.H., Poldrack R.A. (2023). The response time paradox in functional magnetic resonance imaging analyses. Nat. Hum. Behav..

[bib68] Mumford J.A., Bissett P.G., Jones H.M., Shim S., Rios J.A.H., Poldrack R.A. (2024). The response time paradox in functional magnetic resonance imaging analyses. Nat. Hum. Behav..

[bib69] Nilsson H., Rieskamp J., Wagenmakers E.J. (2011). Hierarchical Bayesian parameter estimation for cumulative prospect theory. J. Math. Psychol..

[bib70] Niv Y. (2009). Reinforcement learning in the brain. J. Math. Psychol..

[bib71] Nussenbaum K., Hartley C.A. (2019). Reinforcement learning across development: what insights can we draw from a decade of research. Dev. Cogn. Neurosci..

[bib72] Palminteri S., Kilford E.J., Coricelli G., Blakemore S.-J. (2016). The computational development of reinforcement learning during adolescence. PLOS Comput. Biol..

[bib73] Palminteri S., Wyart V., Koechlin E. (2017). The importance of falsification in computational cognitive modeling.

[bib74] Park S.Q., Kahnt T., Rieskamp J., Heekeren H.R. (2011). Neurobiology of value integration: when value impacts valuation. J. Neurosci..

[bib75] Patzelt E.H., Hartley C.A., Gershman S.J. (2018). Computational phenotyping: using models to understand individual differences in personality, development, and mental illness. Personal. Neurosci..

[bib76] Pedersen M.L., Frank M.J., Biele G. (2017). The drift diffusion model as the choice rule in reinforcement learning. Psychon. Bull. Rev..

[bib77] Peters S., Koolschijn P.C.M.P., Crone E.A., Van Duijvenvoorde A.C.K., Raijmakers M.E.J. (2014). Strategies influence neural activity for feedback learning across child and adolescent development. Neuropsychologia.

[bib78] Poldrack R.A. (2010). Interpreting developmental changes in neuroimaging signals. Hum. Brain Mapp..

[bib79] Poldrack R.A., Baker C.I., Durnez J., Gorgolewski K.J., Matthews P.M., Munafò M.R., Yarkoni T. (2017). Scanning the horizon: towards transparent and reproducible neuroimaging research. Nat. Rev. Neurosci..

[bib80] Rakesh D., Whittle S. (2021). Socioeconomic status and the developing brain – a systematic review of neuroimaging findings in youth. Neurosci. Biobehav. Rev..

[bib81] Rangel A., Camerer C., Montague P.R. (2008). A framework for studying the neurobiology of value-based decision making. Nat. Rev. Neurosci..

[bib82] Ratcliff R., Love J., Thompson C. a, Opfer J.E. (2012). Children are not like older adults: a diffusion model analysis of developmental changes in speeded responses. Child Dev..

[bib83] Rescorla R.A., Wagner A.R. (1972). A theory of Pavlovian conditioning: Variations in the effectiveness of reinforcement and nonreinforcement. Class. Cond.: Curr. Res. Theory.

[bib84] Rodriguez Buritica J.M., Eppinger B., Heekeren H.R., Crone E.A., Van Duijvenvoorde A.C.K. (2024). Observational reinforcement learning in children and young adults. Npj Sci. Learn..

[bib85] Roesch M.R., Esber G.R., Li J., Daw N.D., Schoenbaum G. (2012). Surprise! neural correlates of Pearce-Hall and Rescorla-Wagner coexist within the brain. Eur. J. Neurosci..

[bib86] Rosenblau G., Korn C.W., Pelphrey K.A. (2018). A computational account of optimizing social predictions reveals that adolescents are conservative learners in social contexts. J. Neurosci..

[bib87] Schaaf J.V., Jepma M., Visser I., Huizenga H.M. (2019). A hierarchical Bayesian approach to assess learning and guessing strategies in reinforcement learning. J. Math. Psychol..

[bib88] Schaaf J.V., Xu B., Jepma M., Visser I., Huizenga H.M. (2022). (Mal)Adaptive learning after switches between object-based and rule-based environments. Comput. Brain Behav..

[bib89] Siegler R.S., Opfer J.E. (2003). The development of numerical estimation: evidence for multiple representations of numerical quantity. Psychol. Sci..

[bib90] Sonuga-Barke E.J.S., Cortese S., Fairchild G., Stringaris A. (2016). Annual research review: transdiagnostic neuroscience of child and adolescent mental disorders - differentiating decision making in attention-deficit/hyperactivity disorder, conduct disorder, depression, and anxiety. J. Child Psychol. Psychiatry Allied Discip..

[bib91] Speer S.P.H., Keysers C., Campdepadrós J., Teurlings C.J.S., Smidts A., Boksem M.A.S., Gazzola V. (2023). A multivariate brain signature for reward. NeuroImage.

[bib92] Steingroever H., Jepma M., Lee M.D., Jansen B.R.J., Huizenga H.M. (2019). Detecting strategies in developmental psychology. Comput. Brain Behav..

[bib93] Steingroever H., Wetzels R., Wagenmakers E.-J. (2014). Absolute performance of reinforcement-learning models for the Iowa gambling task. Decision.

[bib94] Teslovich T., Mulder M., Franklin N.T., Ruberry E.J., Millner A., Somerville L.H., Casey B.J. (2014). Adolescents let sufficient evidence accumulate before making a decision when large incentives are at stake. Dev. Sci..

[bib95] Topel S., Ma I., Sleutels J., Van Steenbergen H., de Bruijn E.R.A., Van Duijvenvoorde A.C.K. (2023). Expecting the unexpected: a review of learning under uncertainty across development. Cogn. Affect. Behav. Neurosci..

[bib96] Torrubia R., Avila C., Molto J., Caseras X. (2001). The sensitivity to punishment and sensitivity to reward questionnaire (SPSRQ) as a measure of Gray’s anxiety and impulsivity dimensions. Personal. Individ. Differ..

[bib97] Trepel C., Fox C.R., Poldrack R. a (2005). Prospect theory on the brain? Toward a cognitive neuroscience of decision under risk. Cogn. Brain Res..

[bib98] Turner B.M., Forstmann B.U., Love B.C., Palmeri T.J., Van Maanen L. (2017). Approaches to analysis in model-based cognitive neuroscience. J. Math. Psychol..

[bib99] Turner B.M., Forstmann B.U., Wagenmakers E.J., Brown S.D., Sederberg P.B., Steyvers M. (2013). A Bayesian framework for simultaneously modeling neural and behavioral data. NeuroImage.

[bib100] Turner, B.M., & Mcclure, S.M. (2015). Why more is better: Simultaneous modeling of EEG, fMRI, and behavioral data, (April 2016). ​​​doi:10.1016/j.neuroimage.2015.12.030. 10.1016/j.neuroimage.2015.12.030 26723544

[bib101] Valton, V., Mkrtchian, A., Moses-Payne, M., Gray, A., Kieslich, K., VanUrk, S., … Jonathan Roiser, C. 2024. A computational approach to understanding effort-based decision-making in depression Running Title: Effort-based decision-making in depression, 1–26. Retrieved from doi: 10.1101/2024.06.17.599286. 10.1017/S0033291725101967 PMC12527505 41059633

[bib102] Van Gerven M.A.J. (2017). A primer on encoding models in sensory neuroscience. J. Math. Psychol..

[bib103] Van Maanen L., Miletić S. (2021). The interpretation of behavior-model correlations in unidentified cognitive models. Psychon. Bull. Rev..

[bib104] Venkatraman V., Payne J.W., Bettman J.R., Luce M.F., Huettel S.A. (2009). Separate neural mechanisms underlie choices and strategic preferences in risky decision making. Neuron.

[bib105] Veselic, S., Smid, C.R., Beveridge, F., Steinbeis, N., 2024. Money makes the world go round–but not to the same degree for everyone: developmental changes in specific reward preference..

[bib106] Waltmann M., Herzog N., Reiter A.M.F., Villringer A., Horstmann A., Deserno L. (2023). Diminished reinforcement sensitivity in adolescence is associated with enhanced response switching and reduced coding of choice probability in the medial frontal pole. Dev. Cogn. Neurosci..

[bib107] Westhoff B., Blankenstein N.E., Schreuders E., Crone E.A., Van Duijvenvoorde A.C.K. (2021). Increased ventromedial prefrontal cortex activity in adolescence benefits prosocial reinforcement learning. Dev. Cogn. Neurosci..

[bib108] Williams T.B., Burke C.J., Nebe S., Preuschoff K. (2021). Testing models at the neural level reveals how the brain computes subjective value. PNAS.

[bib109] Wilson R.C., Collins A.G. (2019). Ten simple rules for the computational modeling of behavioral data. eLife.

[bib110] Wilson R.C., Niv Y. (2015). Is model fitting necessary for model-based fMRI. PLOS Comput. Biol..

[bib111] Wittmann B.C., Daw N.D., Seymour B., Dolan R.J. (2008). Striatal activity underlies novelty-based choice in humans. Neuron.

[bib112] Yarkoni T. (2022). The generalizability crisis. Behav. Brain Sci..

[bib113] Zadelaar J.N., Schaaf J.V., Dekkers L.M.S., Agelink van Rentergem J.A., Olthof M.C., Jansen B.R.J., Huizenga H.M. (2021). Development of decision making based on internal and external information: a hierarchical Bayesian approach. Judge. Decis. Mak..

[bib114] Zadelaar J.N., Weeda W.D., Waldorp L.J., Van Duijvenvoorde A.C.K., Blankenstein N.E., Huizenga H.M. (2019). Are individual differences quantitative or qualitative? An integrated behavioral and fMRI MIMIC approach. NeuroImage.

